# Black phosphorus-based biomaterials for temporomandibular joint osteoarthritis: mechanistic rationale, therapeutic potential, and translational considerations

**DOI:** 10.3389/fcell.2026.1992416

**Published:** 2026-10-29

**Authors:** Chaoyi Fang, Leyi Wang, Zhiyi Pan, Gaoli Xu

**Affiliations:** 1 School of Stomatology, Zhejiang Chinese Medical University, Hangzhou, Zhejiang, China; 2 Hospital of Stomatology, Zhejiang Chinese Medical University, Hangzhou, Zhejiang, China

**Keywords:** black phosphorus, fibrocartilage, joint microenvironment, subchondral bone, temporomandibular joint osteoarthritis

## Abstract

**Background:**

Temporomandibular joint osteoarthritis (TMJOA) is a degenerative joint disorder involving coordinated pathological changes in the synovium, condylar fibrocartilage, and subchondral bone. Current treatments primarily relieve symptoms but have limited capacity to modify progressive tissue degeneration. Black phosphorus (BP) combines redox activity, biodegradability, molecular loading capacity, and near-infrared (NIR) responsiveness, making it a potential platform for simultaneously modulating the joint microenvironment and supporting tissue repair.

**Methods:**

We integrated current evidence on the cellular and molecular pathology of TMJOA with experimental studies of BP in osteoarthritis, cartilage repair, and bone regeneration. Particular attention was given to synovial immune regulation, oxidative stress and extracellular matrix homeostasis, fibrocartilage repair, subchondral bone remodeling, NIR-responsive biophysical regulation, and BP-based strategies for stabilization, local delivery, tissue targeting, and osteochondral reconstruction.

**Results:**

TMJOA progression reflects interactions among synovial inflammation, chondrocyte injury, fibrocartilage stem cell (FCSC) dysfunction, abnormal mechanotransduction, and stage-dependent subchondral bone remodeling. BP-based systems can modulate synovial inflammation, reduce oxidative stress and matrix catabolism, and influence bone remodeling. In engineered BP-based systems, mild photothermal stimulation has been reported to affect calcium signaling and mitochondrial function, while material engineering may improve local retention, tissue targeting, and osteochondral repair. However, most evidence derives from knee OA, cartilage-defect, or bone-regeneration models. Direct evidence in TMJOA remains limited, particularly regarding fibrocartilage stem cells, tissue-specific mineralization, osteoblast–osteoclast coupling, and TMJ-specific thermal dosing.

**Conclusion:**

BP provides a multifunctional material platform capable of integrating microenvironmental regulation, local delivery, NIR-responsive control, and osteochondral repair. Current evidence provides a mechanistic rationale for further investigation of BP-based biomaterials in TMJOA, although a disease-modifying effect in the TMJ has not yet been established. Future studies should define material state, local dose, degradation behavior, and NIR exposure in TMJOA-specific models, while determining whether BP-based systems can support FCSC-mediated chondrogenic repair and maintain lineage stability.

## 1 Introduction

Temporomandibular joint osteoarthritis (TMJOA) is a common degenerative disorder of the temporomandibular joint (TMJ), characterized by joint pain, restricted mandibular movement, and progressive structural deterioration of the condyle. Pathological changes extend beyond condylar cartilage to involve the synovium and subchondral bone (Wu et al., 2024). In TMJOA, synovial macrophages and fibroblast-like synoviocytes (FLSs) release inflammatory mediators, including interleukin-1β (IL-1β), tumor necrosis factor-α (TNF-α), and interleukin-6 (IL-6), and modulate chondrocyte function and matrix turnover through extracellular vesicles (EVs), metabolites, and other paracrine signals. Conversely, matrix fragments and damage-associated molecules released from degenerating cartilage can enter the synovial fluid and further activate synovial cells (Wu et al., 2024). With disease progression, persistent synovial inflammation and cartilage matrix loss are accompanied by remodeling of the subchondral bone (Wang et al., 2015).

Cartilage degeneration in TMJOA is closely linked to inflammation, oxidative stress, and abnormal mechanical loading. Excessive reactive oxygen species (ROS) activate inflammatory and catabolic pathways, including nuclear factor-κB (NF-κB) and mitogen-activated protein kinase (MAPK) signaling, thereby promoting chondrocyte apoptosis and extracellular matrix (ECM) degradation (Zhang et al., 2023). Abnormal mechanical loading further perturbs chondrocyte mechanotransduction, Ca^2+^ homeostasis, and metabolic activity, compounding the loss of matrix homeostasis. Unlike joints covered by conventional hyaline cartilage, the mandibular condyle is predominantly fibrocartilaginous and contains fibrocartilage stem cells (FCSCs) in its superficial zone. These resident progenitors have chondrogenic and osteogenic potential and contribute to condylar cartilage maintenance and repair. TMJOA alters FCSC differentiation and is associated with impaired cartilage repair and abnormal subchondral bone remodeling (Li B. et al., 2021; Lu et al., 2022; Bi et al., 2023). Because FCSCs contribute to both chondrogenic and osteogenic lineages, disruption of their self-renewal or lineage specification may affect both fibrocartilage repair and the osteochondral interface.

Current management of TMJOA primarily aims to relieve pain, control inflammation, and improve joint function through conservative treatment, intra-articular interventions, or surgery. Once substantial cartilage destruction and subchondral bone abnormalities have developed, effective disease-modifying options remain limited (Mélou et al., 2023). The confined TMJ space also poses a challenge for local therapy, which must provide sufficient and sustained exposure while addressing pathological changes across the synovium, cartilage, and subchondral bone. These requirements have driven growing interest in biomaterials that combine local delivery, biological activity, and responsiveness to external stimuli (Matheus et al., 2022).

Black phosphorus (BP) is a layered allotrope of phosphorus that can be exfoliated into few-layer phosphorene or black phosphorus nanosheets (BPNSs). Exfoliation increases the exposed surface area and changes oxidation kinetics, while surface functionalization or incorporation into composite matrices further alters surface chemistry and degradation behavior. Its puckered two-dimensional architecture provides a large surface area and abundant chemically accessible sites for loading or conjugating small-molecule drugs, proteins, nucleic acids, and other bioactive components (Bigham et al., 2026). BP has a thickness-dependent direct bandgap and strong near-infrared (NIR) absorption with efficient photothermal conversion, supporting photothermal modulation and stimulus-responsive cargo release (Bigham et al., 2026; Xu et al., 2019). Under physiological conditions, BP gradually degrades into phosphorus-containing products, which contributes to its biodegradability (Bigham et al., 2026). These properties make BP suitable for local therapeutic systems that combine biodegradability, cargo delivery, and NIR responsiveness.

Beyond its role as a carrier and photothermal material, exfoliated BP materials have shown antioxidant effects in experimental OA. Few-layer phosphorene (FLP) catalytically removes ROS, hydrogen peroxide (H_2_O_2_), and nitric oxide (NO), reducing oxidative injury in chondrocytes and limiting the loss of glycosaminoglycans and type II collagen (Zhang et al., 2022). In a separate study, BPNS treatment reduced intracellular ROS and was accompanied by antioxidant enzyme-related transcriptional changes, restoration of chondrocyte function, and regulation of inflammation (Lu et al., 2023). *In vivo*, BPNSs attenuated cartilage degeneration and subchondral bone lesions. These findings support the investigation of BP-derived materials in disorders involving both cartilage and subchondral bone, including TMJOA.

Direct evidence for BP in TMJOA, however, remains limited. Most musculoskeletal studies have focused on knee OA, cartilage injury, or bone regeneration, whereas the TMJ has a fibrocartilaginous articular surface, an articular disc, and a mechanical environment shaped by mastication and occlusion. These joint-specific features warrant direct validation of findings derived from other musculoskeletal models (Sun et al., 2026). This review examines the mechanistic basis for using BP to modulate the pathological microenvironment of TMJOA and assesses its therapeutic potential, biomaterial strategies, and major translational challenges. Table 1 summarizes the experimental context, BP formulation, principal findings, and relevance of the available evidence to TMJOA.

**TABLE 1:** TMJOA pathological targets and supporting evidence from BP-based studies.

Source of evidence	Experimental context	BP formulation/system	Principal findings	Interpretation for TMJOA	Key references
TMJOA-specific mechanistic evidence	Human TMJOA samples and TMJOA experimental models	BP not tested	Synovial inflammation, oxidative stress, abnormal mechanotransduction, FCSC dysfunction, and dysregulated subchondral bone remodeling are central features of TMJOA	Directly defines TMJOA-specific pathological targets, but provides no direct evidence for BP treatment	Bi et al. (2022) ; (2023); Wang et al. (2022b); Shao et al. (2023); Zhen et al. (2023); Li et al. (2024a)
BP in experimental OA	OA chondrocyte models and predominantly knee OA animal models	Few-layer phosphorene (FLP); BP nanosheets (BPNSs)	Reduced oxidative stress and matrix loss, preserved cartilage integrity, and alleviated cartilage degeneration; BPNSs were also associated with improvement of subchondral bone lesions	Provides the closest disease-level therapeutic evidence for extrapolation to TMJOA, although TMJ fibrocartilage and biomechanics differ from those of the knee	Zhang et al. (2022); Lu et al. (2023)
Engineered BP-based systems in OA	Experimental OA models	BPNS–ZnO microspheres; biomimetic BP photothermal systems; macrophage-membrane-coated BPNSs; BPNS-based sustained-release systems	Modulated macrophage responses, reduced synovitis and cartilage damage, and enabled NIR-responsive or sustained delivery	Supports the use of BP as a component of multifunctional OA therapies; effects should be attributed to the complete engineered system rather than BP alone	Lei et al. (2026); Wang et al. (2026b); Yu et al. (2026a) ; (2026b)
Cartilage and osteochondral repair studies	Cartilage injury and osteochondral defect models	WYRGRL-functionalized BPNSs; BP-containing bilayer GelMA scaffolds	Enhanced cartilage targeting and penetration, promoted BMSC chondrogenesis, and improved cartilage–subchondral bone repair	Supports cartilage targeting and osteochondral reconstruction, but focal defect models do not reproduce progressive TMJOA	Dong et al. (2024); Sun et al. (2024a)
Synovial regulation in inflammatory joint disease	Rheumatoid arthritis and inflammatory synovial models	BPNSs	Suppressed pro-inflammatory macrophage activity and altered FLS autophagy and energy metabolism	Provides cross-disease mechanistic support for synovial regulation, but cannot be regarded as direct OA or TMJOA evidence	Zhuang et al. (2024)
Bone remodeling and regeneration studies	Osteoclast differentiation, osteogenesis, biomineralization, and bone-regeneration models	Oxidized BPNSs (oBPNSs); BP; BP quantum dots; BP-containing composites	Inhibited osteoclastogenesis, promoted osteogenic differentiation and mineralization, and modulated osteoimmune responses	Supports potential regulation of subchondral bone remodeling, but these models do not reproduce the stage-dependent bone changes of TMJOA	Tong et al. (2019); Wang et al. (2019); Xu et al. (2021b); Qiu et al. (2024)

## 2 Pathological alterations and cellular mechanisms of TMJOA

### 2.1 Synovial inflammation and the immune microenvironment

#### 2.1.1 Synovial inflammation and cartilage injury

Synovitis is a prominent pathological feature of TMJOA. Sustained inflammatory signaling shifts the intra-articular milieu toward catabolism and promotes ECM degradation (Wu et al., 2024). Shao et al. reported increased levels of high mobility group box 1 (HMGB1), IL-1β, interleukin-18 (IL-18), prostaglandin E2, and other inflammatory mediators in synovial fluid from patients with TMJOA. HMGB1 levels were also associated with pain, mandibular functional limitation, and radiographic disease severity (Shao et al., 2023).

Synovial inflammation disrupts chondrocyte homeostasis by increasing the expression of matrix metalloproteinases (MMPs) and a disintegrin and metalloproteinase with thrombospondin motifs (ADAMTSs), reducing type II collagen and aggrecan, and promoting chondrocyte apoptosis (Zhang et al., 2023; Wu et al., 2024). Conversely, matrix fragments and damage-associated signals released from injured cartilage can reactivate synovial cells and sustain a bidirectional inflammatory loop (Wu et al., 2024).

#### 2.1.2 Functional alterations of synovial macrophages

Macrophages are a major cellular component of the synovial immune microenvironment in TMJOA. Hu et al. reported increased numbers of F4/80-positive synovial macrophages in different TMJOA models, with concomitant increases in both CD86^−^and CD206-positive populations, consistent with the coexistence of distinct macrophage functional states. Non-selective depletion of synovial macrophages failed to ameliorate synovitis and instead aggravated cartilage and subchondral bone damage (Hu et al., 2024). These findings indicate that macrophage function and secretory phenotype, rather than abundance alone, are central to their contribution to TMJOA. Recent work has also implicated M1 macrophage-derived migrasomes in autophagy-related modulation of TMJOA, further illustrating the diversity of macrophage-derived signals (Sa et al., 2026).

Macrophage inflammatory activity is closely linked to intracellular redox status. In lipopolysaccharide (LPS)-activated macrophages and a complete Freund’s adjuvant (CFA)-induced TMJ inflammation model, Zhen et al. showed that nicotinamide adenine dinucleotide phosphate (NADPH) oxidase 4 (NOX4)-dependent ROS production promoted MAPK and NF-κB activation and inflammatory mediator release. NOX4 inhibition reduced intracellular ROS and inflammatory responses and attenuated synovitis and condylar structural damage *in vivo* (Zhen et al., 2023). These data identify NOX4–ROS signaling as a mechanistic link between oxidative stress and inflammatory macrophage activation in the TMJ.

#### 2.1.3 FLSs and synovium–cartilage crosstalk

FLSs are major components of the synovial lining and active regulators of the local inflammatory environment (Han et al., 2020; Wu et al., 2024). In TMJOA, lactate dehydrogenase A (LDHA) is upregulated in synovial tissue and FLSs, together with enhanced glycolytic activity. Pharmacological inhibition of LDHA decreases glucose uptake and lactate production while restoring hyaluronan synthase 2 (HAS2) expression and hyaluronan synthesis (Li et al., 2020).

Beyond FLS-intrinsic metabolic changes, the composition of synovial fluid also varies with disease severity. Zhang et al. identified alterations in multiple amino acid-, lipid-, and cofactor-related metabolites in human TMJOA synovial fluid, with pathways involving cofactor biosynthesis, choline metabolism, mineral absorption, and selenocompound metabolism associated with disease grade (Zhang et al., 2024). Together with altered glycolytic and lactate metabolism in FLSs, these findings broaden synovium–cartilage crosstalk beyond classical cytokines to include metabolites, EVs, and other local signals (Li et al., 2020; Wu et al., 2024; Zhang et al., 2024).

### 2.2 Condylar fibrocartilage injury and impaired repair

#### 2.2.1 Oxidative and metabolic stress–driven ECM catabolism

ROS accumulation in condylar chondrocytes compromises cell survival and ECM homeostasis. Zhang et al. showed that elevated ROS in TMJOA-related chondrocyte models was accompanied by activation of NF-κB and MAPK signaling, inflammatory mediator release, chondrocyte apoptosis, and ECM degradation (Zhang et al., 2023). Reducing excessive ROS attenuated these cellular changes and cartilage damage in experimental TMJOA (Zhang et al., 2023). Related intervention studies also show that suppression of MAPK/NF-κB and AKT/mTOR signaling can preserve cartilage homeostasis in TMJOA (Zhang et al., 2025).

Endogenous antioxidant defenses also influence the response of condylar cartilage to oxidative stress. Xiong et al. showed that nuclear factor erythroid 2-related factor 2 (Nrf2) nuclear translocation and increased heme oxygenase-1 (HO-1) expression reduced ROS accumulation, apoptosis, and IL-1β-induced disruption of ECM metabolism in chondrocytes and condylar cartilage (Xiong et al., 2023). These protective effects were markedly attenuated after Nrf2 knockdown, supporting the Nrf2/HO-1 pathway as an important endogenous antioxidant mechanism in TMJOA.

Metabolic reprogramming can further amplify oxidative stress and matrix catabolism in TMJOA chondrocytes. Glucose-6-phosphate dehydrogenase (G6PD) expression is increased in condylar cartilage, whereas G6PD knockdown reduces NADPH levels, NOX4 expression, and intracellular ROS while suppressing extracellular signal-regulated kinase (ERK), c-Jun N-terminal kinase (JNK), and p38 MAPK phosphorylation and decreasing MMP3, MMP13, and IL-6 expression (Lin et al., 2024). These findings link altered glucose metabolism to ROS production and matrix degradation through the G6PD–NOX4–ROS–MAPK axis.

Pyruvate kinase M2 (PKM2) represents a second metabolic node. Zhao et al. found that ubiquitin-specific peptidase 32 (USP32) was upregulated in TMJOA cartilage and inflammatory chondrocytes and stabilized PKM2 by removing K48- and K11-linked ubiquitin chains. PKM2 accumulation enhanced glycolysis and lactate production and was accompanied by loss of mitochondrial membrane potential, reduced adenosine triphosphate (ATP) production, increased ROS, chondrocyte apoptosis, and ECM degradation (Zhao et al., 2025a). Knockdown of either USP32 or PKM2 partially restored mitochondrial function and attenuated cartilage damage (Zhao et al., 2025a). Related work has also implicated mechanistic target of rapamycin complex 1 (mTORC1)-dependent coordination of autophagy and apoptosis in TMJOA chondrocytes (Yang et al., 2020). Together, these studies link metabolic dysregulation to mitochondrial dysfunction and cell fate in diseased cartilage.

#### 2.2.2 Abnormal mechanotransduction and FCSC repair failure

The TMJ is continuously exposed to complex compressive, shear, and tensile forces, and malocclusion or disc displacement can redistribute mechanical loading across the condyle. Li et al. showed that abnormal mechanical stress increased the expression and activity of piezo type mechanosensitive ion channel component 1 (Piezo1) in chondrocytes, enhanced Ca^2+^ influx, altered the cytoskeleton, and increased catabolic and hypertrophic markers including MMP13, a disintegrin and metalloproteinase with thrombospondin motifs 5 (ADAMTS5), and type X collagen (Li M. et al., 2024). Inhibition of Piezo1 markedly attenuated these responses (Li M. et al., 2024), identifying Piezo1 as one route by which abnormal loading is translated into intracellular Ca^2+^ signaling and catabolic activity. Mechanical stress also engages Wnt/β-catenin signaling: reduced secreted frizzled-related protein expression and increased connexin 43 have each been linked to TMJOA progression (Cai et al., 2022; 2026).

The consequences of abnormal loading also depend on the type of occlusal disturbance. Single-cell transcriptomic analysis comparing anterior premature contact and unilateral anterior crossbite models revealed enhanced communication between chondrocytes and immune cells in both models, but with distinct dominant signaling patterns. TNF-related signaling was more prominent in the former, whereas Thbs1–Sdc4-associated communication was more evident in the latter (Wang K. et al., 2026). These findings indicate that different patterns of mechanical loading can elicit distinct cellular and immune responses within condylar cartilage.

Condylar fibrocartilage retains intrinsic regenerative capacity through FCSCs residing in the superficial zone. FCSCs exhibit clonogenicity, self-renewal, and multilineage differentiation toward chondrogenic and osteogenic lineages and contribute to condylar cartilage homeostasis and repair (Embree et al., 2016; Bi et al., 2020). Human TMJ-derived FCSCs display strong chondrogenic potential, with SRY-box transcription factor 9 (SOX9) playing an important role in maintaining chondrogenic differentiation. SOX9 expression is reduced in the superficial zone of TMJOA condylar cartilage, consistent with impaired FCSC chondrogenic capacity (Bi et al., 2020).

Inflammation can directly compromise this endogenous repair capacity. Bi et al. showed that TNF-α inhibited FCSC proliferation, increased apoptosis, and interfered with both chondrogenic and osteogenic differentiation (Bi et al., 2022). Inhibition of TNF-α/NF-κB signaling partially restored stem cell properties and differentiation capacity and attenuated cartilage degeneration in experimental TMJOA. Thus, injury to mature chondrocytes and dysfunction of endogenous repair cells can occur within the same inflammatory environment.

FCSC fate also varies across TMJOA models and disease contexts. In a disc displacement model, Bi et al. observed impaired chondrogenic differentiation of Gli1^+^ FCSCs together with abnormal osteogenic differentiation of the Sox9^+^ lineage and loss of subchondral bone homeostasis (Bi et al., 2023). By contrast, Wang et al. found that discectomy-induced TMJOA caused excessive activation of Gli1^+^ FCSCs and accelerated their differentiation toward hypertrophic chondrocytes, progressively depleting the stem cell pool (Wang et al., 2024). Selective inhibition of β-catenin in Gli1^+^ cells preserved the FCSC reservoir and alleviated cartilage degeneration (Wang et al., 2024). Both insufficient chondrogenesis and excessive progression toward hypertrophy can therefore compromise the long-term regenerative capacity of condylar fibrocartilage.

### 2.3 Aberrant subchondral bone remodeling

#### 2.3.1 Osteoblast–osteoclast imbalance

Subchondral bone homeostasis depends on coordinated coupling between bone formation and resorption. Under excessive mechanical stress, the activities of both osteoblasts and osteoclasts increase, but osteoclastic activity predominates and is accompanied by structural bone damage (Wang L. et al., 2022). Mechanical stimulation also enhances ERK1/2 signaling in osteoclasts, whereas osteoclast-derived signals promote the osteogenic differentiation of bone marrow mesenchymal stem cells (BMSCs) (Wang L. et al., 2022). Subchondral bone abnormalities are detectable early in experimental TMJOA (Embree et al., 2011), indicating that enhanced bone resorption and compensatory osteogenesis can occur concurrently during remodeling.

Condylar chondrocytes can further promote osteoclastogenesis. Under insufficient occlusal support, TNF-α released by hypertrophic chondrocytes promotes the differentiation of synovium-derived macrophages into osteoclasts and enhances condylar bone resorption; cartilage-specific reduction of TNF-α attenuates both processes (Yan et al., 2024). Osteoclast-derived signals have also been associated with OA pain and degeneration (Zhu et al., 2026), suggesting that osteoclast activity contributes to joint pathology beyond its direct effects on bone resorption.

#### 2.3.2 Subchondral bone remodeling and osteochondral interface alterations

Subchondral bone remodeling in TMJOA is stage-dependent rather than unidirectional. Early stages are often characterized by increased osteoclastic activity, loss of bone mass, and disruption of trabecular architecture, whereas later stages may show reparative bone formation, trabecular thickening, and subchondral sclerosis (Wang L. et al., 2022; Yan et al., 2024; Cardoneanu et al., 2023). Mechanistic studies further indicate that osteogenesis and osteoclastogenesis are not simply sequential events; both can be activated under abnormal mechanical conditions and influence one another through intercellular signaling (Wang L. et al., 2022; Yan et al., 2024). Vascular remodeling accompanies these changes. Transcriptomic and endothelial-cell studies have identified coupled inflammatory–angiogenic signaling and OX40 ligand (OX40L)-dependent angiogenesis and osteoclastogenesis in experimental TMJOA (Qin et al., 2024; Zheng Y. et al., 2026).

FCSC lineage dysregulation provides a cellular bridge between condylar cartilage and subchondral bone. In disc displacement-induced TMJOA, impaired chondrogenic differentiation of Gli1^+^ FCSCs occurs together with abnormal osteogenic differentiation of the Sox9^+^ lineage and loss of subchondral bone homeostasis (Bi et al., 2023). Superficial-zone stem/progenitor cell fate is therefore relevant not only to cartilage renewal but also to maintenance of the osteochondral interface.

Single-cell transcriptomic studies further support extensive multicellular communication across the condylar osteochondral unit. In malocclusion-induced TMJOA, distinct chondrocyte and immune-cell populations exhibit enhanced intercellular signaling, with communication networks varying among mechanical models (Wang K. et al., 2026). Together with osteoclast activation and altered FCSC fate, these findings indicate that subchondral remodeling is shaped by the combined effects of mechanical loading, immune signaling, and cross-tissue cellular interactions.

Changes in trabecular architecture, subchondral plate thickness, and mineralization alter load transmission and can, in turn, affect the overlying fibrocartilage (Wang L. et al., 2022; Bi et al., 2023; Cardoneanu et al., 2023; Yan et al., 2024; Wang K. et al., 2026). This cross-tissue feedback connects synovial inflammation, fibrocartilage injury and impaired repair, and subchondral bone remodeling, providing a pathological basis for treating these compartments as an interconnected therapeutic unit.

TMJOA can be viewed as the convergence of four interrelated pathological processes: synovial inflammatory and immune dysregulation, oxidative and metabolic injury of condylar fibrocartilage, abnormal mechanotransduction with impaired FCSC-mediated repair, and stage-dependent subchondral bone remodeling. These processes are linked through several recurrent signaling axes, including NOX4–ROS–MAPK/NF-κB, Nrf2/HO-1, G6PD–NOX4–ROS–MAPK, USP32–PKM2, AKT/mTOR, Piezo1–Ca^2+^, Wnt/β-catenin, TNF-α/NF-κB, and ERK1/2. Rather than acting independently, these pathways connect inflammatory, metabolic, mechanical, and regenerative responses across the synovium, fibrocartilage, and subchondral bone. The major cellular and molecular interactions are summarized in Figure 1.

**FIGURE 1:**
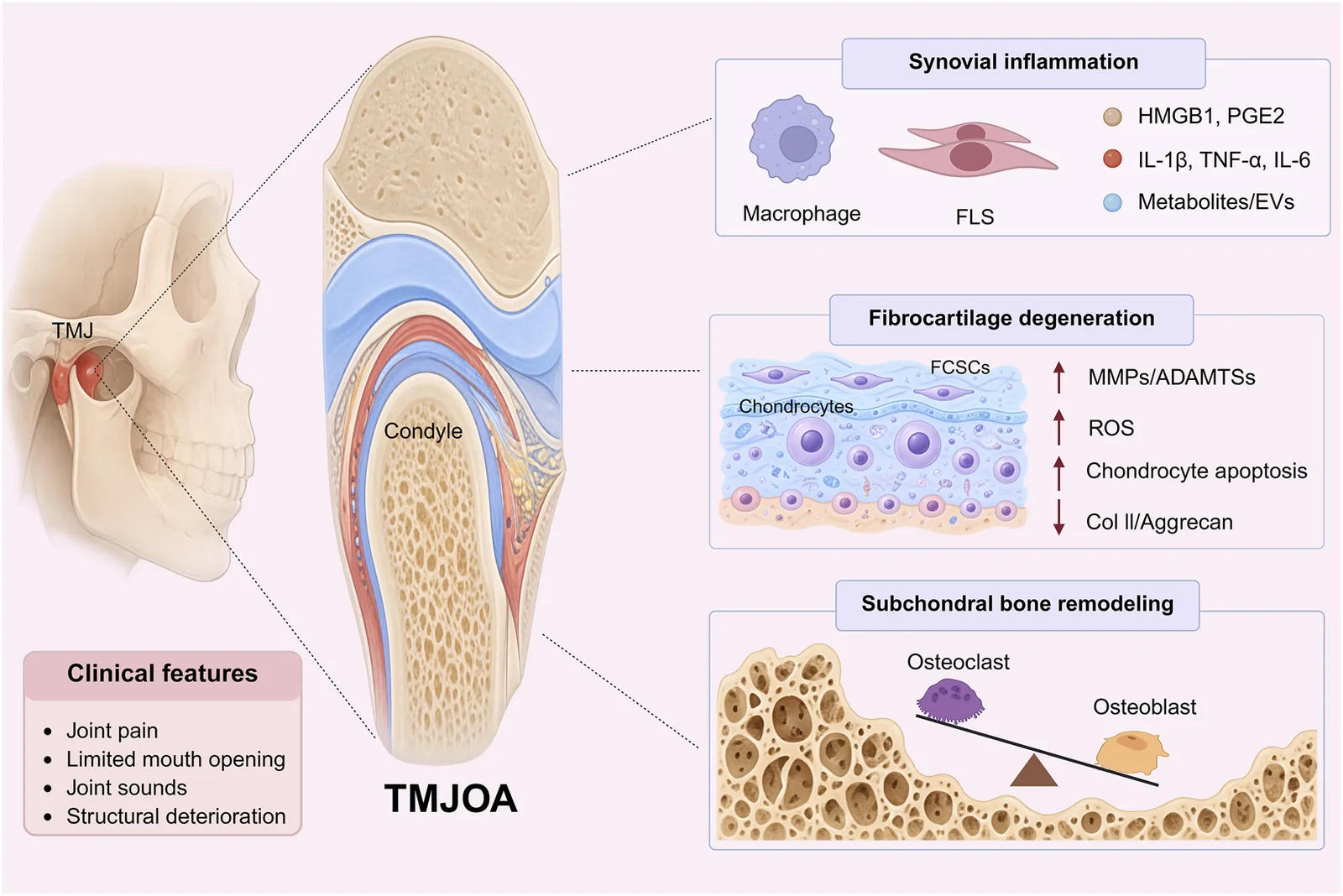
Pathological alterations and cellular mechanisms involved in TMJOA. Schematic illustration of the major pathological processes underlying TMJOA, including synovial inflammation, condylar fibrocartilage degeneration, and subchondral bone remodeling. The figure highlights the interactions among immune cells, fibroblast-like synoviocytes, chondrocytes, fibrocartilage stem cells, and bone cells during disease progression.

## 3 Cellular, molecular, and biophysical mechanisms of BP in modulating the joint microenvironment

BP is chemically unstable in aqueous, oxygen-containing environments. Surface oxidation progressively disrupts the phosphorus lattice and shortens material persistence, while continued oxidation generates phosphorus-containing degradation products. Phosphate released during degradation can participate in calcium phosphate mineralization, which may support subchondral bone regeneration but may also favor unwanted mineral deposition in fibrocartilage. Rapid degradation can additionally shorten the period over which BP retains its photothermal and cargo-delivery functions.

BP also shows strong NIR absorption and efficient photothermal conversion, enabling externally controlled heating and stimulus-responsive cargo release. These functions provide temporal control over local treatment, although excessive thermal exposure may damage cartilage and adjacent tissues. Its layered structure additionally offers a large surface area for loading drugs, proteins, nucleic acids, and other bioactive molecules, while aggregation and instability in physiological media remain practical limitations. The principal advantage of BP therefore lies not in any single biological effect, but in its ability to combine redox regulation, biodegradability, molecular loading, and NIR responsiveness within one material platform. This combination provides a mechanistic basis for coordinated intervention across synovial inflammation, fibrocartilage degeneration and repair, and subchondral bone remodeling.

### 3.1 Synovial inflammation and immune regulation

#### 3.1.1 Modulation of macrophage functional states

Macrophage responses have been investigated using several BP-containing therapeutic systems in inflammatory joint models. Yu et al. developed a biomimetic nanosystem containing BPNSs (Yu et al., 2026a). In OA synovium, treatment decreased CD86-positive macrophages and increased CD206-positive macrophages, with concurrent reductions in synovial hyperplasia and cartilage degeneration. These findings show that the biomimetic BP-containing nanosystem modulates macrophage phenotype and attenuates synovial inflammation in experimental OA.

Lei et al. reported a similar immune effect using BPNS–ZnO composite microspheres (Lei et al., 2026). Treatment suppressed NF-κB-associated inflammatory signaling and pro-inflammatory mediator expression while increasing macrophage phenotypes associated with inflammation resolution. *In vivo*, these changes coincided with reduced synovitis, better preservation of cartilage proteoglycans, and less structural joint damage. Because BPNSs and ZnO were combined in this system, their individual contributions cannot be separated; nevertheless, macrophage regulation was a clear component of the overall response.

BPNSs themselves can also act directly on macrophages. In a rheumatoid arthritis model, Zhuang et al. showed that inflammatory macrophages internalized BPNSs, with reduced expression of TNF-α and IL-6 and increased expression of the repair-associated markers interleukin-10 (IL-10) and arginase 1 (ARG1) (Zhuang et al., 2024). *In vivo*, these changes were accompanied by less synovial hyperplasia, local inflammation, and structural joint damage. Despite differences in BP formulations and disease models, the common response across these studies was reduced pro-inflammatory macrophage activity together with a shift in functional state (Zhuang et al., 2024; Lei et al., 2026; Yu et al., 2026a). The biological interface can further modify this response: protein-corona formation on BPNSs altered macrophage polarization through Ca^2+^-dependent signaling (Mo et al., 2020). Although this effect has not been tested in OA or TMJOA, it indicates that adsorbed proteins may influence BP–immune cell interactions.

A consistent feature across these studies is that BP-based interventions alter macrophage function rather than simply reducing macrophage abundance. This distinction is relevant to TMJOA, where macrophages with different functional states coexist in the synovium and non-selective macrophage depletion can aggravate synovitis, cartilage injury, and subchondral bone damage (Hu et al., 2024). Modulation of macrophage state may therefore be more relevant to BP-mediated immune regulation than depletion of the total macrophage population.

#### 3.1.2 FLSs and synovium–cartilage crosstalk

FLSs are another major cellular component of the inflamed synovium. Zhuang et al. found that BPNSs were internalized by inflammatory FLSs and altered both autophagy and energy metabolism (Zhuang et al., 2024). BPNS treatment was associated with changes in AMP-activated protein kinase/mammalian target of rapamycin (AMPK/mTOR)-related signaling, suppression of aberrant autophagy, and a shift from aerobic glycolysis toward oxidative phosphorylation. ATP production increased, whereas inflammation- and senescence-associated molecules declined. This metabolic pattern is mechanistically relevant to the elevated LDHA expression, enhanced glycolysis, and altered lactate metabolism reported in TMJOA FLSs (Li et al., 2020).

Macrophage regulation can also propagate to cartilage and synovial stromal cells through paracrine signaling. Yu et al. cultured chondrocytes with conditioned media from differently treated macrophages (Yu et al., 2026a). Media from macrophages exposed to the BP-based system increased type II collagen expression and reduced MMP13 and TNF-α in chondrocytes. When applied to FLSs, the same conditioned media reduced fibrosis-associated markers, including type I collagen, type III collagen, and α-smooth muscle actin, together with migratory and invasive activity.

TMJOA synovium–cartilage crosstalk involves inflammatory mediators, metabolites, and EVs (Li et al., 2020; Wu et al., 2024; Zhang et al., 2024). The available BP studies extend this concept by showing that changes in macrophage secretory activity and FLS function can influence neighboring chondrocytes. BP-mediated control of synovial inflammation may therefore involve both direct effects on synovial cell populations and secondary effects transmitted to cartilage through intercellular communication.

### 3.2 Cartilage protection and repair

#### 3.2.1 ROS scavenging and cellular antioxidant responses

Zhang et al. prepared few-layer phosphorene (FLP) from bulk BP by ultrasound-assisted mechanical exfoliation and demonstrated efficient elimination of ROS, H_2_O_2_, and NO in OA chondrocytes (Zhang et al., 2022). FLP reduced intracellular oxidative stress and improved chondrocyte viability. *In vivo*, intra-articular administration of FLP preserved glycosaminoglycans and type II collagen and attenuated cartilage matrix damage. These findings support the ROS- and reactive nitrogen species (RNS)-scavenging activity of FLP in the OA microenvironment.

BPNSs have shown a similar reduction in oxidative stress. Lu et al. reported that BPNS treatment markedly decreased intracellular ROS in OA chondrocytes and preserved the balance between cartilage matrix anabolism and catabolism (Lu et al., 2023). Ribonucleic acid (RNA) sequencing further revealed antioxidant enzyme-related transcriptional changes, restoration of chondrocyte function, and regulation of inflammation. These transcriptomic changes indicate a cellular antioxidant response accompanying the reduction in intracellular ROS. *In vivo*, BPNS treatment reduced cartilage degeneration and improved subchondral bone lesions.

These antioxidant effects are relevant to TMJOA, where excessive ROS contributes to chondrocyte apoptosis, ECM degradation, metabolic dysregulation, and mitochondrial dysfunction (Xiong et al., 2023; Zhang et al., 2023; Lin et al., 2024; Zhao et al., 2025a). However, current evidence does not establish direct regulation of upstream metabolic pathways, including the G6PD–NOX4–ROS–MAPK and USP32–PKM2 axes, by FLP or BPNSs.

#### 3.2.2 ECM turnover and maintenance of the chondrocyte phenotype

The cartilage response to BP also extends to ECM turnover. FLP reduced the loss of glycosaminoglycans and type II collagen in OA cartilage (Zhang et al., 2022). After BPNS treatment, transcriptional programs related to chondrocyte function and ECM homeostasis were improved, while *in vivo* tissue showed better matrix preservation and less surface damage.

Lei et al. provided more detailed molecular evidence using BPNS–ZnO composite microspheres (Lei et al., 2026). In inflamed chondrocytes, treatment reduced the matrix-degrading mediators MMP13, MMP3, and ADAMTS5 and increased type II collagen, aggrecan, and SOX9. Proteoglycan 4 (PRG4), a key contributor to articular-surface lubrication, was also restored. *In vivo*, lower MMP13 and ADAMTS5 and higher PRG4 were accompanied by improved proteoglycan preservation and cartilage surface integrity (Lei et al., 2026). The overall response combined reduced matrix catabolism with preservation of anabolic and lubrication-associated chondrocyte phenotypes.

These matrix-level effects converge with several pathways already implicated in TMJOA. G6PD/NOX4-related metabolic abnormalities increase MMP3 and MMP13 expression (Lin et al., 2024), whereas abnormal loading through Piezo1–Ca^2+^ signaling promotes MMP13 and ADAMTS5 (Li M. et al., 2024). BP-based treatments improve several of these downstream matrix markers (Zhang et al., 2022; Lu et al., 2023; Lei et al., 2026), but the available evidence does not establish direct regulation of the upstream metabolic or mechanosensitive pathways.

#### 3.2.3 Stem/progenitor cell responses and cartilage repair

In focal cartilage-defect models, repair depends in part on stem/progenitor cells with chondrogenic potential. Dong et al. functionalized BPNSs with the type II collagen-binding peptide WYRGRL to generate cartilage-targeting BPNSs (BP-W) (Dong et al., 2024). BP-W preferentially localized to damaged cartilage, penetrated the cartilage matrix, and promoted chondrogenic differentiation of BMSCs. In a mouse knee-cartilage injury model, these effects were accompanied by improved defect repair and tissue regeneration.

For osteochondral repair, chondrogenic and osteogenic responses must also be coordinated across the cartilage and subchondral bone compartments. Sun et al. fabricated a three-dimensional (3D)-printed bilayer gelatin methacryloyl (GelMA) scaffold in which the upper layer contained BP and human umbilical cord mesenchymal stem cell-derived exosomes and had a relatively low elastic modulus to support BMSC chondrogenesis (Sun et al., 2024a). β-Tricalcium phosphate (β-TCP) was incorporated into the lower layer to increase mechanical strength and promote osteogenesis. In a rabbit osteochondral defect model, the construct improved repair of both cartilage and subchondral bone, providing distinct microenvironments for chondrogenic and osteogenic repair within the osteochondral unit.

Condylar fibrocartilage contains a resident stem/progenitor population with particular relevance to TMJOA. FCSCs reside mainly in the superficial zone and exhibit clonogenicity, self-renewal, and both chondrogenic and osteogenic potential, contributing to condylar cartilage homeostasis and repair (Embree et al., 2016; Bi et al., 2020). TMJOA alters FCSC maintenance and lineage behavior. TNF-α/NF-κB signaling impairs FCSC proliferation and differentiation, while reduced SOX9 expression is associated with diminished chondrogenic capacity (Bi et al., 2020; 2022). In disc displacement-induced TMJOA, impaired chondrogenic differentiation of Gli1^+^ FCSCs occurs together with abnormal osteogenic differentiation of the Sox9^+^ lineage and loss of subchondral bone homeostasis (Bi et al., 2023). In discectomy-induced TMJOA, excessive activation of Gli1^+^ FCSCs promotes hypertrophic differentiation and progressive depletion of the stem-cell population, whereas inhibition of β-catenin helps preserve FCSC function and attenuate cartilage degeneration (Wang et al., 2024).

Current BP-based cartilage-repair studies involving stem/progenitor cells have focused mainly on BMSCs, whereas effects on TMJ FCSCs have not yet been directly demonstrated. This is particularly relevant because FCSC fate contributes to both fibrocartilage repair and osteochondral homeostasis. Future studies should therefore determine whether BP-based systems can maintain FCSC self-renewal and chondrogenic capacity without accelerating hypertrophic or osteogenic differentiation under the inflammatory and abnormal mechanical conditions of TMJOA. Relevant endpoints include clonogenicity, SOX9 expression and chondrogenic differentiation, hypertrophic differentiation, and osteogenic lineage commitment.

### 3.3 Regulation of subchondral bone remodeling

#### 3.3.1 Osteogenic differentiation and mineralization

BP activity within the joint also extends to the subchondral compartment. In an OA model, Lu et al. showed that BPNSs reduced cartilage degeneration while promoting osteogenesis; micro-CT and histology also showed improvement in subchondral bone lesions (Lu et al., 2023). These joint-level findings are consistent with the broader osteogenic potential reported across BP-based bone-regeneration systems (Cheng et al., 2020).

Cell-based osteogenic studies have used several BP formulations. Jeon et al. examined ultrathin BP layers, whereas Li et al. and Xu et al. incorporated BP into collagen- or hydrogel-based constructs; these formulations increased alkaline phosphatase (ALP) activity, mineral deposition, and osteogenic marker expression in preosteoblasts or mesenchymal stem cells (Jeon et al., 2021; Li L. et al., 2021; Xu H. et al., 2021). In ectomesenchymal stem cells, BP nanoparticles also promoted osteogenic differentiation through upregulation of transglutaminase 2 (TG2) (Lu et al., 2021). Although these studies used different BP formulations and cell systems, they consistently support progression toward an osteogenic phenotype.

The consequences of these osteogenic effects may differ between FCSCs and bone-forming progenitors. FCSCs contribute to fibrocartilage homeostasis and retain both chondrogenic and osteogenic potential. In TMJOA, abnormal osteogenic differentiation within the FCSC lineage has been associated with disruption of condylar osteochondral homeostasis (Bi et al., 2023). BP-based materials should therefore be evaluated separately in FCSCs and subchondral bone progenitors, as enhanced osteogenesis may support bone repair but be undesirable within the fibrocartilage stem-cell compartment.

BP degradation provides a second route to mineral formation. Under physiological conditions, BP is progressively oxidized and generates phosphate-containing products. Inorganic phosphate can participate in calcium phosphate deposition together with Ca^2+^ (Wang et al., 2019; Sun et al., 2024b). Wang et al. incorporated BP quantum dots into bioinspired matrix vesicles and showed that BP-derived phosphate promoted mineral deposition, cellular mineralization, and new bone formation (Wang et al., 2019). Additional hydrogel studies indicate that BP-associated osteogenesis can vary with sustained phosphate availability, particle size, and matrix formulation (Huang et al., 2019; Miao et al., 2019; Xu H. et al., 2022). Thus, the phosphorus composition and degradability of BP contribute to mineralization in addition to its effects on osteogenic differentiation.

Osteogenesis can also be coupled to immune and vascular regulation. Qiu et al. found that BP increased interleukin-33 (IL-33) expression in macrophages (Qiu et al., 2024). Co-culture of BP-treated macrophages with BMSCs increased runt-related transcription factor 2 (RUNX2), bone morphogenetic protein 2 (BMP2), collagen type I (COL1), and osteocalcin (OCN), together with ALP activity and mineralization; loss of Il-33 weakened these responses and reduced bone repair *in vivo*. In non-joint bone-regeneration models, BP-containing deoxyribonucleic acid (DNA) hydrogels, 3D-printed scaffolds, and deferoxamine-containing hydrogels have also coupled osteogenesis with vascular or osteoimmune support (Xu D. et al., 2022; Long et al., 2023; Miao et al., 2023). Although these models do not reproduce TMJOA, they broaden the mechanistic context of BP-mediated bone repair beyond direct effects on osteogenic cells.

These bone-regenerative effects require a different interpretation in TMJOA, where subchondral bone remains in a state of abnormal turnover rather than a simple healing response. Mechanical overload can increase osteoclastic activity early in disease while compensatory bone formation occurs in parallel; later stages may show trabecular thickening and subchondral sclerosis (Wang L. et al., 2022; Cardoneanu et al., 2023; Yan et al., 2024). Osteogenic and mineralization responses to BP, BPNSs, and BP-containing composites should therefore be evaluated together with bone resorption and changes in bone microarchitecture.

#### 3.3.2 Osteoclast regulation and osteoblast–osteoclast balance

Oxidized BP can also act directly on osteoclasts. Xu et al. showed that oxidized black phosphorus nanosheets (oBPNSs) inhibited receptor activator of nuclear factor-κB ligand (RANKL)-induced osteoclast differentiation. oBPNSs reduced multinucleated osteoclast formation, actin-ring formation, and bone-resorptive activity without marked cytotoxicity (Xu Y. et al., 2021). They also suppressed p38 MAPK and NF-κB signaling and reduced nuclear factor of activated T cells 1 (NFATc1), a key regulator of osteoclastogenesis.

TMJOA provides a pathological context in which this antiresorptive effect may be relevant. Abnormal mechanical loading increases osteoclast formation and bone resorption in condylar subchondral bone, and activated osteoclasts can in turn influence BMSC osteogenic differentiation through intercellular signaling (Wang L. et al., 2022). Under insufficient occlusal support, TNF-α released by hypertrophic chondrocytes promotes differentiation of synovium-derived macrophages into osteoclasts and aggravates condylar bone resorption (Yan et al., 2024). Osteoclasts therefore link altered mechanics and cartilage abnormalities to subchondral bone remodeling.

Osteogenic studies have used ultrathin BP layers, BP nanoparticles, BPNSs, and BP-containing composites (Jeon et al., 2021; Li L. et al., 2021; Xu H. et al., 2021; Qiu et al., 2024), whereas the direct antiresorptive study used oxidized BPNSs (oBPNSs) (Xu Y. et al., 2021). Oxidation alters the BP surface and accompanies phosphate release, so antiresorptive findings obtained with oBPNSs should not be extrapolated directly to unoxidized BP formulations.

TMJOA subchondral bone can exhibit increased resorption, compensatory bone formation, and later sclerosis within the same disease course. No study has yet evaluated BP effects on bone formation and resorption simultaneously in a TMJOA model. Whether BP can normalize bone turnover and osteoblast–osteoclast coupling in the mandibular condyle therefore remains unresolved.

### 3.4 NIR-responsive biophysical regulation

#### 3.4.1 NIR-triggered material responses

NIR absorption is a core physicochemical feature of BP. Functionalized BP nanomaterials retain strong NIR absorption and efficiently convert light into heat, although photothermal output varies with material concentration, surface structure, and incident energy (Tong et al., 2019; Bigham et al., 2026; Yu et al., 2026a; 2026b). In biomimetic BP systems, cell-membrane coating did not markedly reduce photothermal performance, and stable heating was maintained over repeated irradiation–cooling cycles (Yu et al., 2026a; 2026b).

NIR irradiation also changes the behavior of BP-based materials. Shao et al. showed that NIR exposure accelerated conversion of BP to PO_4_^3-^ and promoted mineral deposition through reactions between PO_4_^3-^ and Ca^2+^; the location and extent of mineralization varied with irradiation site and duration (Shao et al., 2020). In poly (lactic-co-glycolic acid) (PLGA) microspheres containing BPNSs and SrCl_2_, local photothermal heating disrupted the polymer shell and triggered Sr^2+^ release (Wang X. et al., 2018). NIR irradiation can therefore regulate degradation, ion release, and mineralization in addition to generating heat.

#### 3.4.2 Mild photothermal regulation of cellular responses

At non-ablative temperatures, photothermal heating generated by engineered BP-based systems can act as a biophysical signal rather than a cytotoxic insult. Yu et al. showed that mild heating generated by an engineered BP-based photothermal system reduced transient receptor potential vanilloid 4 (TRPV4) activity in macrophages, with lower Ca^2+^ influx and mitochondrial Ca^2+^ overload (Yu et al., 2026a). Mitochondrial membrane potential, basal respiration, ATP production, and maximal respiratory capacity were improved.

Changes in TRPV4-dependent Ca^2+^ signaling were accompanied by a shift in macrophage function. Photothermal treatment with this engineered BP-based system reduced CD86 and inducible nitric oxide synthase (iNOS) and increased mannose receptor C-type 1 (MRC1) and ARG1. Signal transducer and activator of transcription 6 (STAT6) nuclear translocation and early growth response 2 (EGR2) activity also increased (Yu et al., 2026a). Conditioned medium from these macrophages promoted ECM synthesis in chondrocytes and reduced fibrosis-related matrix expression in FLSs, linking the macrophage response to downstream effects on neighboring joint cells.

A macrophage-membrane-coated BPNS system showed a similar pattern. NIR treatment further reduced CD86 and iNOS while increasing CD206 and ARG1. Conditioned medium from treated macrophages increased proteoglycan and type II collagen in chondrocytes, reduced MMP13 and ADAMTS5, and suppressed the fibrotic and migratory phenotype of FLSs (Yu et al., 2026b).

The cellular response to mild BP-mediated heating is context dependent. Tong et al. found that periodic NIR stimulation of BPs@PLGA induced heat shock proteins (HSPs) and enhanced osteogenic differentiation and bone formation *in vivo* (Tong et al., 2019). In synovial macrophages, photothermal regulation is linked to ion flux and mitochondrial metabolism, whereas in osteogenic cells a heat-shock response contributes to bone formation.

#### 3.4.3 Thermal dose and tissue response

Biological responses to BP-mediated photothermal treatment depend on the delivered thermal dose, which reflects wavelength, power density, exposure time, material concentration, and the temperature reached in the target tissue. Most current BP studies use an 808 nm NIR laser, but energy input and resulting temperature profiles vary among material systems.

Yu et al. used 100 μg/mL of the BP-based material and 808 nm irradiation at 1.25 W/cm^2^ to maintain a temperature of approximately 42 °C for mechanistic experiments. In another biomimetic BPNS system, 808 nm irradiation at 0.75 W/cm^2^ for 180 s increased temperature by about 6 °C; *in vivo* treatment used three 180-s irradiation periods separated by 30-s intervals and was repeated once weekly (Yu et al., 2026a; 2026b). A BP-based photothermal hydrogel reached a stable temperature of 42 °C ± 1 °C after 5 min of irradiation at 808 nm and 1 W/cm^2^ (Zhang et al., 2026).

These studies illustrate that the same wavelength does not produce an equivalent thermal exposure across BP formulations. Photothermal conversion efficiency, BP concentration, composite structure, and tissue coverage all influence the temperature–time profile. Power density and exposure duration should therefore be interpreted together with the measured tissue temperature.

Mild BP-mediated photothermal studies commonly operate near 40 °C–42 °C (Tong et al., 2019; Yu et al., 2026a; Zhang et al., 2026), but this range derives mainly from *in vitro* and non-TMJ animal experiments and should be regarded as an experimental reference rather than an established TMJ treatment window. Data on repeated heating of condylar cartilage, synovium, the articular disc, and adjacent tissues remain limited. A TMJ-specific thermal dose–response window will require direct measurement of joint temperature, exposure duration, and local tissue responses. The cellular, molecular, and biophysical mechanisms of BP-mediated regulation are summarized in Figure 2.

**FIGURE 2:**
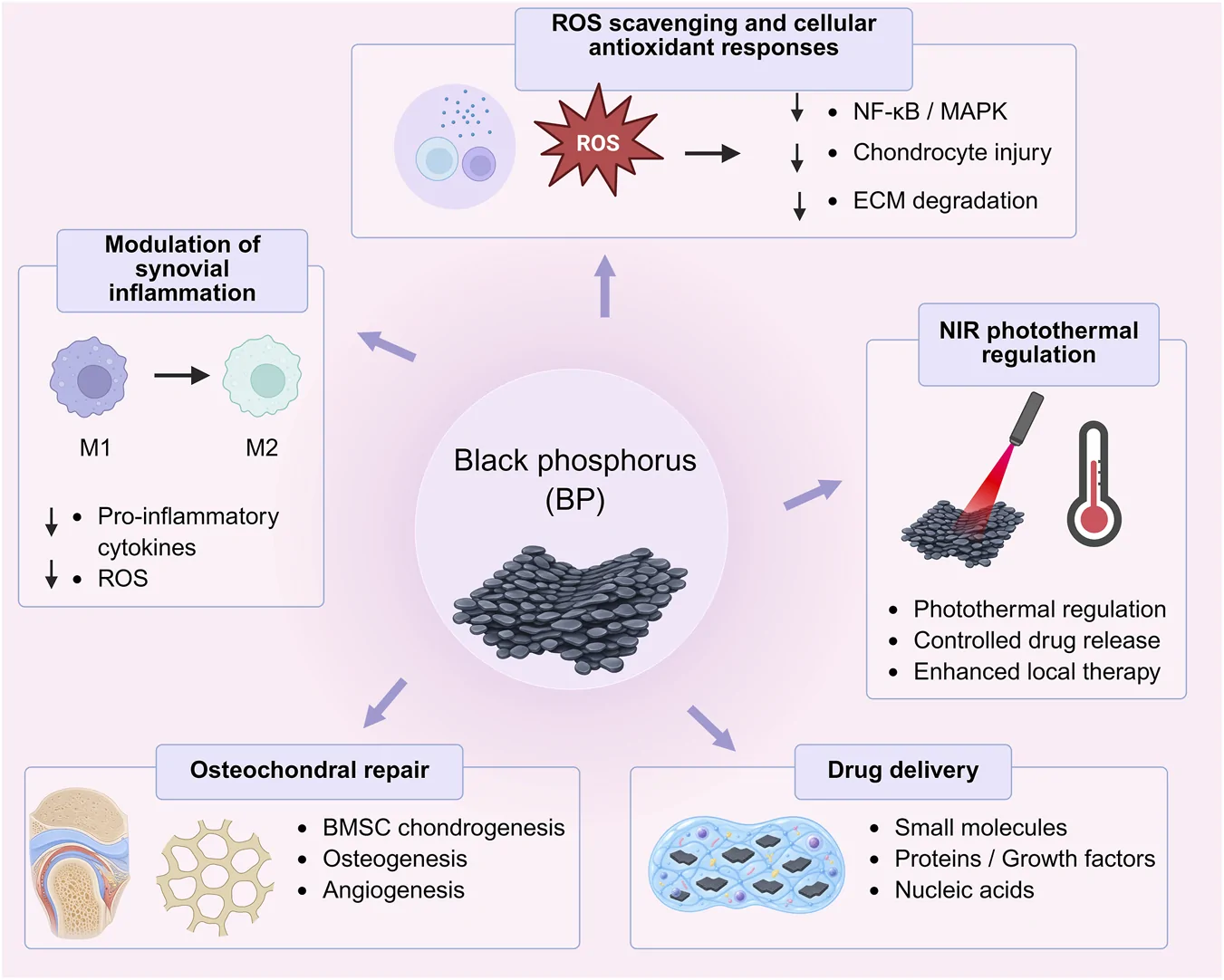
Cellular and molecular effects of BP and BP-based systems relevant to the TMJOA microenvironment. The schematic summarizes the major cellular and molecular effects of BP and BP-based systems relevant to the TMJOA microenvironment, including modulation of synovial inflammation, ROS scavenging and cellular antioxidant responses, attenuation of chondrocyte injury and ECM degradation, osteochondral repair, NIR-responsive photothermal regulation, and drug delivery.

## 4 BP-based therapeutic strategies and their applicability to TMJOA

### 4.1 Stabilization and functionalization of BP

#### 4.1.1 Regulation of oxidation and degradation

Environmental stability is a central design constraint for BP-based local therapy. The lone-pair electrons of surface phosphorus atoms make BP susceptible to oxidation in the presence of oxygen, water, and light, with progressive changes in surface chemistry and crystal structure that can reduce photothermal performance, cargo loading, and functional lifetime (Abellán et al., 2017; Bigham et al., 2026). Degradation kinetics also depend on nanosheet thickness, lateral size, and surface state. Abellán et al. showed that thinner BP nanosheets oxidize more rapidly, while lateral dimensions and light exposure further influence degradation (Abellán et al., 2017). Limiting oxygen and water access through size and thickness control, surface passivation, polymer coating, or carrier encapsulation can prolong functional stability under physiological conditions (Abellán et al., 2017; Tao et al., 2017; Zeng et al., 2018; Zeng and Chen, 2020). Coordination-based surface protection and small-molecule passivation provide additional routes to stabilize BP without eliminating its layered structure (Zhao et al., 2016; Shi et al., 2020).

For TMJOA, the objective is not maximal persistence but controlled functional stability. A locally delivered BP system should retain sufficient structural integrity and photoresponsiveness during treatment, yet remain degradable after its intended function is completed. This balance is particularly relevant in the confined TMJ space. Phosphate released during BP degradation can contribute to calcium phosphate deposition and bone mineralization (Wang et al., 2019; Sun et al., 2024b), but TMJOA subchondral bone undergoes stage-dependent remodeling, with early bone loss and resorption followed by compensatory formation and sclerosis in later disease (Wang L. et al., 2022). Phosphate release should therefore be interpreted in relation to the local remodeling state rather than assumed to be uniformly beneficial. Conversely, overly rapid degradation may shorten the effective window for NIR stimulation or cargo release. Functional lifetime, oxidation and phosphate-release kinetics, and their relation to dosing and irradiation schedules are therefore more informative than material stability alone.

#### 4.1.2 Surface modification and biomimetic functionalization

Surface modification can improve BP dispersion, slow oxidation, and provide reactive sites for drug, biomolecule, or targeting-ligand conjugation. Tao et al. showed that amino-terminated polyethylene glycol (PEG) markedly reduced BPNS aggregation in phosphate-buffered saline and cell culture medium, with good dispersion maintained after 1 week (Tao et al., 2017). Polydopamine (PDA) coating likewise improved environmental stability and photothermal performance while providing catechol and amino groups for further functionalization (Zeng et al., 2018). More broadly, covalent and noncovalent surface chemistries can tune BP aggregation, degradation, and biointerface interactions (Zeng and Chen, 2020). Peptide passivation and covalent alkoxy modification offer additional means of improving stability and solubility (Wang H. et al., 2018; Zhao et al., 2021). BP-containing hydrogels also show that stabilization within a composite matrix can be coupled to osteogenic function (Wu et al., 2025), although evidence from bone-defect models does not establish joint-specific performance.

Biomimetic coatings and inorganic components can further tailor how BP interacts with joint tissues. Yu et al. used M2 macrophage membranes to coat a BPNS-containing photothermal system; the construct retained its NIR response while modulating synovial macrophages and inflammation in OA (Yu et al., 2026a). Lei et al. combined BPNSs with ZnO in injectable polysaccharide microspheres, coupling redox regulation with effects on macrophages and chondrocytes (Lei et al., 2026). For TMJOA, functionalization should follow the intended tissue target. Macrophage-directed interfaces are most relevant to synovitis, whereas cartilage-directed systems require adequate matrix binding and penetration. This distinction is important because BP can also promote osteogenesis and mineralization, while FCSCs in TMJOA may undergo abnormal hypertrophic or osteogenic differentiation (Bi et al., 2023; Wang et al., 2024). Cartilage-focused BP formulations should therefore be evaluated for hypertrophy and ectopic mineralization in addition to conventional repair endpoints.

### 4.2 Local delivery and tissue-targeting strategies

#### 4.2.1 Hydrogel-based local retention and delivery

Intra-articular injection places therapeutic materials directly within the diseased joint, but local administration alone does not ensure sustained exposure. Free drugs and nanomaterials may be cleared through synovial fluid exchange or taken up by synovial cells, shortening their effective residence time. For BP, rapid clearance can limit redox activity, cargo delivery, and NIR-responsive functions. Incorporation into hydrogels or microspheres can reduce nanosheet diffusion and provide more controlled retention and release.

OA studies provide proof of concept for this approach. Lei et al. incorporated BPNS–ZnO nanocomposites into injectable polysaccharide microspheres and reported reduced synovial inflammation and cartilage damage after intra-articular delivery (Lei et al., 2026). Wang et al. loaded platelet-derived growth factor-BB (PDGF-BB) onto BPNSs and encapsulated the complex in chitosan microspheres (Wang X. et al., 2026). The system released PDGF-BB for more than 5 months, and NIR irradiation further modulated the release profile; a single intra-articular dose produced prolonged effects in a mouse OA model. These studies support BP-containing carriers as a means of extending local exposure while retaining stimulus-responsive release.

TMJOA studies also support the use of injectable local matrices, even though most do not yet contain BP. A tilapia type I gelatin/hyaluronic acid (HA) hydrogel reduced inflammation, improved matrix metabolism, and promoted condylar cartilage repair in rats (Xu and Sun, 2025). Other TMJOA systems, including a PEG-based NELL-1 hydrogel and a ROS-responsive hydrogel, likewise improved local cartilage repair and inflammatory outcomes (Wang C. et al., 2022; Kuang et al., 2025), while broader OA work has emphasized pathology-responsive hydrogel design (Zheng Z. et al., 2026). These studies support the carrier concept but do not define how BP-loaded hydrogels behave in the TMJ. Direct comparison of free and hydrogel-encapsulated BP should therefore include intra-articular residence, cargo-release kinetics, NIR-induced temperature profiles, treatment duration, and material integrity under masticatory loading.

#### 4.2.2 Cartilage- and synovium-targeted delivery

Joint retention and cartilage penetration are distinct delivery problems. Articular cartilage is avascular and contains a dense ECM, which restricts nanoparticle diffusion into deeper regions, while intra-articular particles can also be sequestered by synovial cells and macrophages. Prolonged joint residence therefore does not guarantee effective cartilage delivery. Targeting ligands or affinity-based surfaces can increase interactions with selected matrix components or cell populations and improve tissue-specific exposure. Cartilage-targeted hydrogel microspheres have further shown that selective localization can enhance local lubrication and protection in damaged cartilage (He et al., 2024).

Cartilage-targeted BP has been demonstrated directly in a knee injury model. Dong et al. functionalized BPNSs with the type II collagen-binding peptide WYRGRL to generate BP-W (Dong et al., 2024). Compared with unmodified BPNSs, BP-W showed greater localization to injured cartilage, deeper tissue penetration, enhanced BMSC chondrogenesis, and improved defect repair. Its targeting depends on binding to the α1 chain of type II collagen. Whether the same strategy performs similarly in TMJOA is unresolved because condylar fibrocartilage differs from knee hyaline cartilage in matrix organization and cellular composition, and ECM degradation progresses with disease (Bi et al., 2023; Zhang et al., 2023; Wu et al., 2024). Binding, penetration depth, and retention of BP-W across condylar cartilage zones have not yet been defined.

TMJOA studies have already shown that synovium and cartilage can be targeted separately. Yang et al. used folate-modified nanoparticles to deliver microRNA-330–3p (miR-330–3p) to inflammation-associated macrophages and WYRGRL-modified nanoparticles to deliver kartogenin (KGN) to cartilage (Yang et al., 2024). Combined treatment reduced synovial inflammation and condylar cartilage degeneration in rats. A comparable dual-targeting strategy has not yet been tested with BP. Its value should be assessed by comparing targeted and nontargeted BP for synovial and cartilage accumulation, macrophage uptake, cartilage penetration, effective dose, therapeutic response, and local safety.

### 4.3 Stratified osteochondral repair and interface design

#### 4.3.1 Stratified structure and tissue-specific material design

The TMJ condyle is organized as a stratified osteochondral unit in which superficial fibrocartilage transitions into deeper cartilage and subchondral bone. These regions differ in ECM composition, cell phenotype, and mechanical function, so restoration of zonal organization is an important component of condylar repair. Yu et al. used a bilayer scaffold with segregated surface wettability to promote fibrocartilage-like and hyaline cartilage-like phenotypes in different regions, producing layered repair in goat condylar defects (Yu et al., 2021). Wang et al. combined a HA/type I collagen hydrogel with biphasic calcium phosphate to support cartilage and subchondral bone repair in rabbits (Wang et al., 2021). Complementary TMJ work using superwettable GelMA-MSC microspheres further supports the influence of local matrix properties on condylar cartilage regeneration (Yang et al., 2022).

More recent work has reproduced finer zones within the condyle. Fu et al. designed a trilayer scaffold in which aligned nanofibers supported a fibrocartilage-like superficial region, micron-scale fibers promoted deeper cartilage formation, and a hydroxyapatite-rich layer supported subchondral bone (Fu et al., 2026). The three regions regenerated distinct tissue phenotypes *in vivo*. These studies show that spatial control of surface architecture, mineral content, and mechanical properties can direct region-specific osteochondral repair.

BP has already been incorporated into stratified osteochondral constructs. Sun et al. developed a bilayer 3D-printed GelMA scaffold in which a relatively soft BP/exosome-containing cartilage layer supported BMSC chondrogenesis, whereas β-TCP in the subchondral layer increased stiffness and promoted osteogenesis (Sun et al., 2024a). The construct improved cartilage and subchondral bone repair in rabbits. Related BP hydrogels combined with demineralized bone matrix, gene-silencing components, or mineralized chitosan have also enhanced osteogenic repair in bone-defect settings (Li Z. et al., 2024; Xu et al., 2026). Together, these studies support the use of BP within regionally distinct material phases, while TMJ-specific osteochondral constructs still require direct evaluation.

#### 4.3.2 Gradient interface and mechanical matching

The condylar osteochondral unit is mechanically graded rather than separated by an abrupt cartilage–bone boundary. Su et al. identified a progressive increase and densification of hydroxyapatite toward subchondral bone, accompanied by a gradual change in interface mechanics (Su et al., 2023). Gologorsky et al. likewise reported marked depth-dependent differences in cartilage shear properties (Gologorsky et al., 2021). Together with the distinct mechanical role of the superficial fibrous zone (Ruggiero et al., 2015; Chandrasekaran et al., 2021), these findings support preserving a continuous transition from fibrocartilage to bone in TMJ repair constructs.

From a materials perspective, this transition can be approximated by graded changes in polymer composition, mineral content, pore architecture, or modulus (Zhang et al., 2020; Nordberg et al., 2024). This is directly relevant to BP-based constructs because BPNS content can itself alter hydrogel mechanics (Xu H. et al., 2021). BP concentration should therefore be tuned together with the surrounding matrix rather than treated only as a bioactive additive. Mechanical evaluation can focus on compression and shear behavior, interlayer bonding, and stability under cyclic loading alongside cartilage and bone regeneration endpoints. Representative BP-based biomaterial and local delivery strategies are summarized in Figure 3.

**FIGURE 3:**
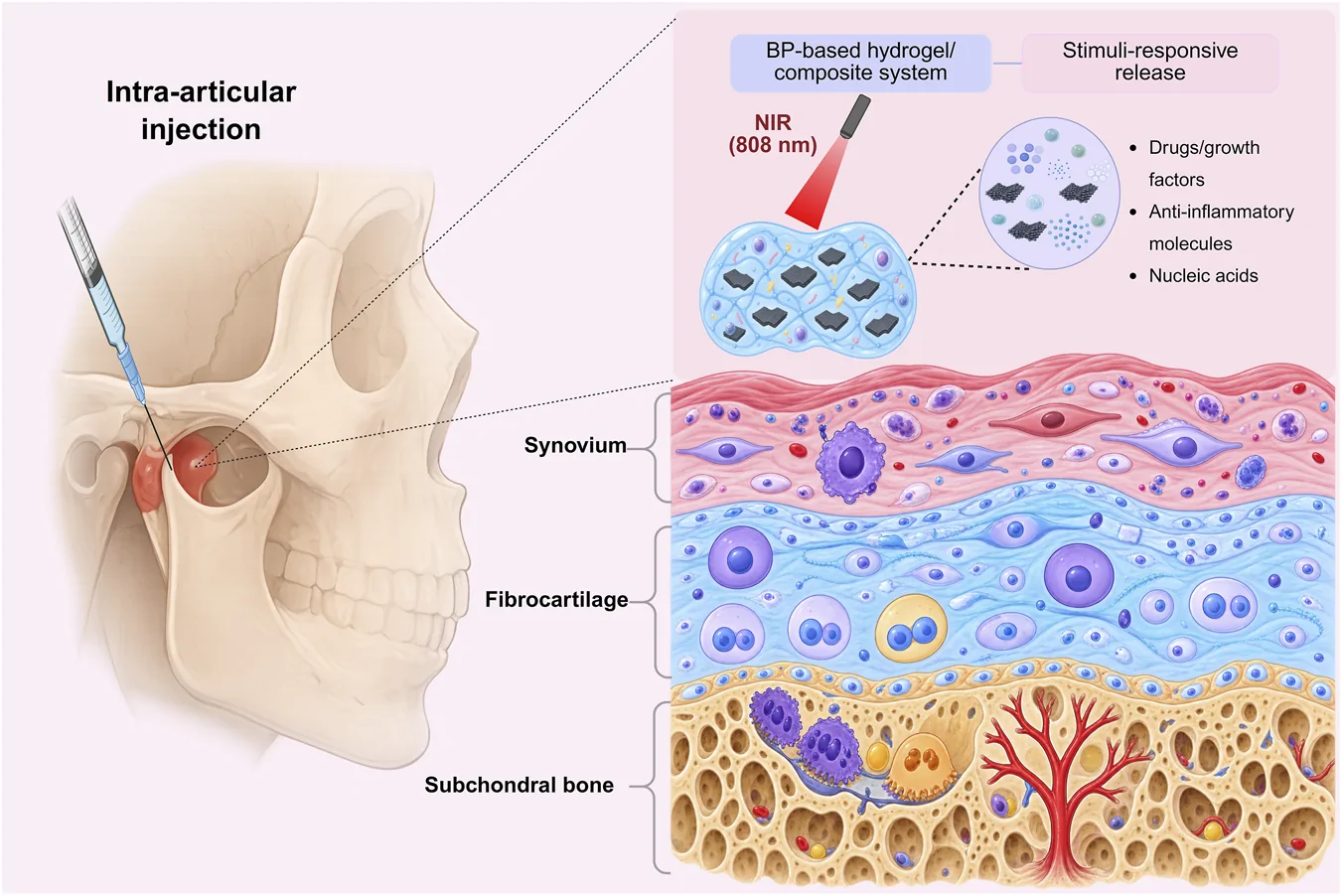
BP-based biomaterial strategies with potential applications in TMJOA. Schematic representation of potential BP-based biomaterial strategies for TMJOA, including intra-articular injection, BP-based hydrogel/composite systems, NIR-responsive release, and local delivery of therapeutic agents. These approaches are intended to enhance local retention and controlled release within the joint and to support coordinated regulation of the synovium, fibrocartilage, and subchondral bone.

### 4.4 Applicability of BP-Based therapeutic strategies to TMJOA

Most BP studies in joint disease derive from knee OA, cartilage injury, or osteochondral repair, whereas direct evidence in TMJOA remains sparse. Although knee OA and TMJOA share synovial inflammation, cartilage degeneration, and subchondral remodeling, they differ in cartilage phenotype, mechanical environment, injury pattern, and pathological calcification (Zhao et al., 2025b). Knee models therefore provide an important experimental basis, but material parameters and treatment effects cannot be transferred to the TMJ without direct validation.

The cartilage target itself is also different. The knee is primarily covered by hyaline cartilage, whereas the mandibular condyle contains a fibrous–hyaline structure with distinct superficial and deep zones (Chandrasekaran et al., 2021). FCSCs in the superficial zone contribute to tissue maintenance and repair (Embree et al., 2016; Bi et al., 2020). WYRGRL-functionalized BPNSs improve localization and penetration in injured knee cartilage by binding type II collagen, but type II collagen is concentrated more deeply in condylar cartilage. Whether the same targeting strategy reaches the relevant TMJ zones is unknown. Likewise, chondrogenic effects of BP-containing systems have been studied mainly in BMSCs (Dong et al., 2024; Sun et al., 2024a), leaving their effects on FCSC self-renewal and lineage stability unresolved. Direct testing in primary FCSCs or lineage-traced FCSC models is therefore needed to determine whether BP-containing systems preserve self-renewal and chondrogenic capacity under inflammatory and mechanically abnormal conditions. Relevant endpoints should include FCSC pool maintenance, clonogenicity, chondrogenic differentiation, hypertrophic transition, and osteogenic lineage bias.

Joint mechanics impose a second constraint. The TMJ combines rotation and translation and is continuously shaped by occlusal and neuromuscular loading; tensile loading is also more prominent than in the knee (Zhao et al., 2025b). Depth-dependent mechanical heterogeneity (Gologorsky et al., 2021) and the load-bearing, lubricating role of the superficial fibrous zone (Ruggiero et al., 2015) may influence how locally delivered materials behave during mastication. Retention and release of injectable carriers should therefore be measured under TMJ-relevant conditions. For NIR-responsive BP systems, irradiation settings from other tissues or knee OA models may guide initial experiments, but thermal dose should ultimately be defined by local TMJ temperature and tissue response.

Mineralization also requires tissue-specific interpretation. BP degradation releases phosphate that can promote bone mineralization (Wang et al., 2019), yet pathological calcification is a recognized feature of TMJOA and differs from knee OA in distribution and morphology (Zhao et al., 2025b). In TMJ models, abnormal mechanical loading induces early mineral deposition (Zhang et al., 2016), alters exosome-associated mineralization regulators such as tissue-nonspecific alkaline phosphatase (TNAP), ectonucleotide pyrophosphatase/phosphodiesterase 1 (NPP1), and matrix Gla protein (MGP) (Liu et al., 2022), and is attenuated by reducing phosphorus intake (Duan et al., 2021). BP-derived phosphate may therefore support subchondral bone repair while posing a different risk in condylar fibrocartilage. Cartilage mineralization and subchondral bone remodeling should be evaluated separately and interpreted together with matrix integrity, hypertrophic change, and bone microarchitecture.

Model context is equally important. Most BP-based osteochondral materials have been tested in focal defect models, which primarily measure tissue regeneration after a defined structural injury (Sun et al., 2024a). TMJOA models instead retain ongoing disease drivers such as inflammation, disc pathology, and altered mechanical loading. Available models—including intra-articular injection, surgical intervention, and abnormal loading—produce different pathological patterns and time courses (Zhao et al., 2022), while disc displacement, perforation, and discectomy differ in severity and progression (Zhou et al., 2023). The model should therefore match the intended mechanism of the BP system. Synovial immunomodulation requires meaningful assessment of synovitis; osteochondral remodeling requires preservation of both cartilage and subchondral bone pathology; and NIR-responsive systems should compare BP alone with BP plus irradiation within the same disease model.

Current OA and osteochondral repair studies provide a strong experimental basis for BP-mediated cartilage protection, local delivery, and tissue regeneration. Whether these effects translate to TMJOA will depend on the fibrous cartilage architecture, mechanical environment, and mineralization pattern of the TMJ. Direct validation in disease models that reproduce TMJ-specific pathology remains essential.

## 5 Discussion

TMJOA progression reflects coordinated pathological changes across the synovium, condylar fibrocartilage, and subchondral bone. Within this interconnected tissue environment, BP is of interest because its biological effects and material properties act on several processes implicated in disease progression. Across OA and tissue-regeneration models, BP-based systems modulate inflammatory macrophage and FLS activity, reduce oxidative injury and matrix catabolism, and influence bone formation, resorption, and mineralization (Xu Y. et al., 2021; Qiu et al., 2024; Zhuang et al., 2024; Lei et al., 2026; Yu et al., 2026a). Material engineering can further enhance these effects by improving stability, intra-articular retention, tissue targeting, and NIR-responsive control. The combination of biological activity and material tunability is particularly relevant to a disease in which inflammatory, cartilaginous, and osseous abnormalities are closely linked.

Evidence for this concept is nevertheless derived mainly from knee OA, cartilage defect, and bone-regeneration models. In OA models, FLP and BPNSs have been reported to reduce oxidative injury and preserve cartilage or subchondral bone (Zhang et al., 2022; Lu et al., 2023), while WYRGRL-functionalized BPNSs and BP-containing bilayer constructs have been used for cartilage targeting and osteochondral repair (Dong et al., 2024; Sun et al., 2024a). The TMJ differs from the knee in fibrocartilage composition, FCSC biology, osteochondral architecture, mastication-related loading, and mineralization patterns (Zhao et al., 2025b). These differences may affect ligand accessibility, material retention, thermal exposure, and the biological consequences of phosphate release. The current literature therefore supports TMJOA-specific investigation, but does not yet establish a disease-modifying effect of BP in the TMJ.

Among the unresolved questions, FCSC biology is particularly relevant to cartilage repair. Chondrogenic responses to BP-containing systems have been studied mainly in BMSCs (Dong et al., 2024; Sun et al., 2024a), whereas TMJOA alters FCSC maintenance and lineage behavior (Bi et al., 2022; 2023; Wang et al., 2024). Whether BP preserves the FCSC pool or modifies these pathological lineage shifts remains unknown. Subchondral bone presents a second issue because remodeling is stage dependent, with resorption, compensatory formation, and sclerosis occurring across the disease course (Cardoneanu et al., 2023; Yan et al., 2024). Osteogenic studies have used BP, BPNSs, or BP-containing composites, whereas direct antiresorptive activity has been demonstrated with oBPNSs; phosphate release has been examined in separate degradation and biomineralization models. Pathological mineralization of condylar cartilage must also be distinguished from reparative mineralization in subchondral bone (Duan et al., 2021). NIR responsiveness adds a further TMJ-specific question. Abnormal loading activates Piezo1–Ca^2+^ signaling, whereas mild photothermal stimulation delivered by an engineered BP-based system was reported to modulate TRPV4-related Ca^2+^ signaling (Li M. et al., 2024; Yu et al., 2026a). Whether these mechano- and thermo-sensitive pathways interact, and how such interactions influence efficacy and safety, remains unresolved.

An important translational question is whether BP-based systems can support tissue repair without disrupting FCSC fate. In diseased condylar fibrocartilage, a favorable response would involve preservation of the FCSC pool and restoration of chondrogenic repair while limiting hypertrophic or ectopic osteogenic differentiation. These stem-cell outcomes should be evaluated together with synovial inflammation, mechanical loading, and subchondral bone remodeling, all of which influence FCSC behavior during TMJOA progression (Bi et al., 2022; 2023; Wang et al., 2024).

Future evaluation of BP should move beyond isolated antioxidant or regenerative endpoints and determine whether its multiple actions can be coordinated in TMJOA models that retain synovial inflammation, fibrocartilage degeneration, and subchondral bone remodeling. Material state, local dose, degradation profile, and NIR exposure need to be assessed alongside tissue-specific responses and clinically relevant joint-level outcomes, including pain-related behavior and mandibular function. Future studies should establish these relationships to determine whether the mechanistic versatility of BP translates into consistent therapeutic effects in the TMJ and to define the conditions required for safe and effective local treatment. Key translational considerations and future directions are summarized in Figure 4.

**FIGURE 4:**
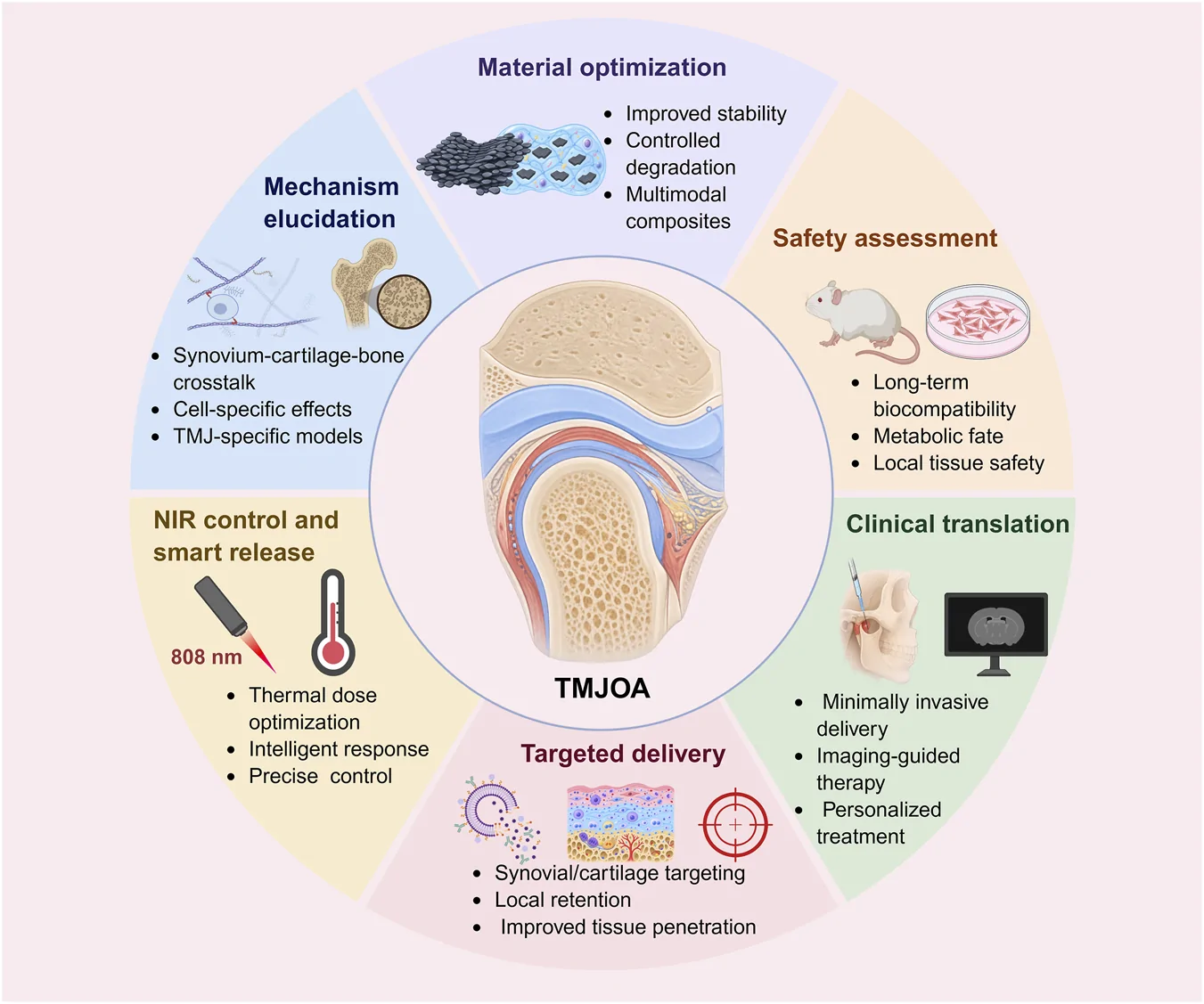
Translational considerations and future directions for BP-based strategies in TMJOA. The schematic summarizes the major translational considerations and future directions for BP-based strategies in TMJOA, including mechanism elucidation in TMJ-specific models, material optimization, targeted delivery, NIR-responsive control and smart release, safety assessment, and clinical translation.
